# Activity Profiles among Older Adults: Latent Class Analysis Using the Korean Time Use Survey

**DOI:** 10.3390/ijerph18168786

**Published:** 2021-08-20

**Authors:** Yungsoo Lee

**Affiliations:** Department of Social Welfare, Incheon National University, 119 Academy-ro, Yeonsu-gu, Incheon 22012, Korea; yslee@inu.ac.kr

**Keywords:** older adults, activity in later life, KTUS, LCA, time use

## Abstract

This study empirically explored the activity profiles of Korean older adults by considering a wide range of activities simultaneously and further investigated the socioeconomic factors associated with activity profiles. Gender differences in activity profiles were examined in-depth. Latent class analysis (LCA) was used to identify activity profiles based on a nationally representative sample of older adults from the most recent two waves of the Korean Time Use Survey (*n* = 3034 for 2014 and *n* = 3960 for 2019). Multinomial logistic regression analysis was employed to further examine the factors associated with the activity profiles. The findings revealed four distinct activity groups, although there were differences in activity profiles between the two waves. Several sociodemographic factors, such as gender, age, assets and income, were significantly associated with the activity profiles. Findings from this study can inform policy makers seeking interventions that enhance the overall well-being of older adults through activity engagement.

## 1. Introduction

Population aging has been occurring rapidly throughout the world, and Korea is not an exception. The proportion of older adults aged 65 or above was 14.9% in 2019, classifying Korea as an aged society [1]. According to the estimates from Statistics Korea (2019), Korea will become a super-aged society by as early as 2026, when the proportion of older adults reaches 20% of the total population. Rapid population aging poses challenges to policy makers as well as researchers, and attention has increasingly been paid to the concept of “active” or “productive” aging. Active aging can be defined as the process of optimizing opportunities for health, participation and security to enhance quality of life as people age, and the term “active” captures a broad range of activities, including participation in social, economic, cultural, spiritual and civic affairs, not just participation in the labor force [2,3]. Productive aging refers to older people’s participation in various activities that contribute to generating goods or services or developing the capacity to produce goods or services [4,5,6]. While these two concepts differ in several ways [7], they share common aspects such as active engagement and participation in a wide range of activities.

Older adults are faced with changing roles and relationships in the family and society, which may narrow their scope for social engagement and limit opportunities for engaging in activities. However, old age can be a productive period of life, depending on the approaches one takes, as well as how one confronts and engages with this phase of life [8]. The literature has confirmed that active participation and engagement in productive activities not only enhance the well-being of older adults but also produce value for families and society as a whole [9]. The literature on active and productive aging places emphasis on a wide range of activities, such as paid work, volunteering, caregiving, informal helping, grand-parenting, enjoying leisure time and engaging in physical activity. Empirical research has shown that these activities are closely linked to various well-being outcomes, including but not limited to physical and mental health, cognitive function and mortality [10,11,12,13,14,15,16].

Activities that older adults engage in on a daily basis are at the core of the literature; however, prior studies are not free from limitations. Many studies tend to focus on a single or limited number of activity domains, such as physical activity [14,17], informal and formal social activity [18,19], solitary or sedentary behavior [19] and leisure activity [17,18,20]. Other researchers have focused directly on productive activity engagement in later life, such as paid work, caregiving and volunteering [21,22,23,24,25,26]. While these studies contribute to the literature on activity and well-being outcomes in later life, they often ignore the reality that most people engage in multiple activities simultaneously on a daily basis, and a series of activities can compete with and complement each other.

Research has begun focusing on a broader range of activities simultaneously [27,28,29,30,31]; however, empirical evidence is still limited, especially for the population of Korean elderly adults. There are a few studies that have examined activity engagement among Korean older adults. For example, Chung and her colleague empirically analyzed the patterns of time use across age and gender using latent class analysis (LCA) [32]. While this study classified activity groups based on a series of activity items in the multiple waves of the KTUS, they only presented time use patterns of the dominant three domains of activities, paid work, education and relationship/leisure, across classes for the simplicity of comparisons. Another study examined time use patterns of the Korean elderly based on k-means cluster analysis focusing on four activity domains: Paid work, household management, care/support activities and self-development [33]. Other studies based on the sample of Korean older adults have also suffered from limitations in that they used time use data that was rather old or focused on a single or limited number of activities [34,35].

To fill the gap in the extant literature, this study empirically examined the activity profiles of Korean older adults by applying LCA to the most recent two waves of the Korean Time Use Survey (KTUS). We utilized a wide range of activities captured from daily time use data to identify activity profiles, and activity items utilized in the analysis encompass not just paid work, social participation and caregiving, but also leisure activity, physical activity, interior and exterior household chores and other things. This study further explored which individual and socioeconomic characteristics were associated with activity profiles derived from the LCA with a focus on gender disparities in activity profiles. It has been widely reported that activity engagement between genders may vary by country depending on the social norms and cultural contexts of gender roles [36,37]. Given the fact that traditional gender roles are still prevalent, especially among the elderly population in Korea [38,39], it is worthwhile to take gender dimensions into account.

## 2. Literature Review

### 2.1. Relating Activity Domains to One Another: Theories and Evidence

The important question is whether older adults can simultaneously engage in different domains of activities and how those are related to one another. There are at least two competing theoretical perspectives: The role-substitution (or strain) perspective and the role-enhancement (or extension) perspective. The role-substitution perspective posits that there are potential tradeoffs between activities given the time and effort constraints one faces [40]. This perspective emphasizes tradeoffs between paid work and other activities based on the assumption that paid work is time demanding and difficult to combine with other activities [40,41,42]. Due to the loss of a role from paid work after retirement, paid work is substituted for other activities such as volunteering or caregiving. In contrast, the role-enhancement perspective emphasizes potential complementarities among activities, suggesting that one domain of activity could lead to more active engagement in other domains of activities [40,43]. Activities in one domain may facilitate opportunities for engaging with others by enhancing the social resources and networks in which individuals are embedded [29].

Empirical research has not provided consistent evidence on the two competing perspectives. It has been reported that a substitution of roles between paid work and volunteering or between paid work and caregiving could exist [22,44,45]. Empirical evidence on the relationship between caregiving and volunteering is more mixed. A few studies have reported a positive relationship between caregiving and volunteering [46], supporting the role-enhancement perspective; however, other studies have confirmed a negative relationship between caregiving and other activities, suggesting that the burden of and time constraints from caregiving could be an obstacle to engaging in other activities [47]. More recently, Morrow-Howell and her colleagues showed that productive activities could be substituted for paid work, but there could be complementarities among productive activities except for paid work [15].

A relatively new and growing body of research has emphasized the role of macro-level factors in activity engagement, such as policy contexts and social norms. For example, Stanfors and colleagues showed that time spent on caregiving negatively influenced time spent on paid work among older adults in the United Kingdom and Canada, but this was not the case for the Swedish elderly population [48]. They concluded that cross-national differences may be due to the well-known social infrastructure for caregiving in Sweden.

In sum, there are two opposite theoretical perspectives on how different domains of activities are related to one another and the empirical evidence has not provided consistent findings. Additionally, these relationships may vary by the social, cultural and policy contexts in which each country or society is embedded. Therefore, it is still important to add empirical evidence on the activity profiles of older adults in Korean contexts.

### 2.2. Gender Dimensions in Activity Profiles

This study takes gender dimensions into account and investigates how activity profiles vary by gender. A traditional gender-role model suggests that men and women may engage in different combinations of activity domains [49]. It is well known that there are gender differences in terms of paid work and caregiving. In many countries and societies, women exit the labor market early to engage in caregiving [50]. Women are more likely to invest time in caregiving than men are and women continue their caregiving roles into old age [51]. Empirical evidence has supported the hypothesis that there is a negative relationship between caregiving and paid work for elderly women, but this often does not hold for elderly men [52]. Another study based on a representative sample of American older adults showed that although a certain type of caregiving (e.g., spousal caregiving) was negatively associated with formal and informal volunteering, this negative relationship existed only for female older adults, lending partial support for the role-substitution or strain perspective [47]. Female older adults are less active in leisure and other activities than their male counterparts [53], at least partly due to the family burdens they face.

Gender disparities in activity engagement are shaped and influenced by social norms and cultural contexts. According to Burda and her colleagues, time distributions between paid and nonpaid work vary by country depending on the social norms and cultural contexts for gender roles [37]. Based on analyses of older adults across nine high-income countries, Gauthier and Smeeding found substantial variation in terms of time use patterns. A large fraction of the time freed up by retirement was reallocated to passive activities and gender differences existed, although they tended to decrease with age [54].

While still limited, a few studies have empirically explored gender disparities in activity engagement among the Korean elderly population. For example, female older adults in Korea spent approximately 2 to 4 times more time on housework and caregiving than their male counterparts [55]. Park [34] also examined the time use of Korean elderly adults based on the 1999 and 2004 Korean Time Use Survey (KTUS) waves, focusing on gender differences in time use for paid and unpaid work and leisure. She found that it was primarily elderly women who engaged in unpaid work (e.g., household chores and caregiving.), leading to higher total working times and much lower leisure times than for elderly men.

While gender disparities in activity engagement among older adults have been widely reported across many countries and societies, they could be even starker in Korean contexts considering the prevalent social norms and cultural contexts for gender roles in Korea [38,39]. An empirical analysis of activity profiles based on a representative sample of the Korean elderly that takes gender dimensions into account may provide important evidence on this issue.

## 3. Materials and Methods

### 3.1. Data

To empirically identify activity profiles, we utilized the Korean Time Use Survey (KTUS) 2014 and 2019. The KTUS includes a nationally representative sample of over 26,000 within approximately 12,000 households. Questions included in the KTUS focus primarily on time use on a wide range of activities based on the time diary method. It is well known that the time diary method provides higher quality estimates of activities than surveys based on stylized questions about amounts of time [56,57]. The survey also covers data on individual-level socioeconomic characteristics such as age, gender, self-rated health, education, income and so on.

The sample in the current study was restricted to older adults aged 65 or above. Since there could be differences between older adults living in urban and rural areas in terms of activities and the contexts related to them, this study only included older adults living in urban areas. In the KTUS, each respondent was asked to fill out time diaries structured in 10-min intervals for two consecutive days. Considering potential differences between activities during weekdays and weekends, this study only included respondents who filled out time diaries on weekdays. Additionally, data for the second day were included in the subsequent analysis because research has shown that the second day data are more accurate [58]. The final sample sizes for the current analysis are 3034 for 2014 and 3960 for 2019, respectively.

### 3.2. Measurement

The KTUS 2014 classified 138 daily activities into nine main categories, which were further divided into 42 subcategories [59]. The KTUS 2019 classified 153 daily activities into same nine main categories, which were further divided into 45 subcategories [60]. Following the method used by Morrow-Howell and her colleagues [15], this study allocated activity items into eight activity domains, and those eight activity domains were utilized in the LCA. Morrow-Howell and her colleagues originally developed nine activity domains based on content analysis and exploratory and confirmatory factor analysis. Activity domains in the current study are slightly different considering differences in the activity items and sample compositions between the KTUS and the HRS. For example, one activity domain, managing medical conditions, was excluded from this study because this category did not add analytic value [15].

Details of the domains and activity items utilized in this study are presented in Table 1. Each activity item was originally measured continuously (e.g., minutes per day) and activity items in each domain were summed and recoded into three-level ordinal measures representing low (or no), medium and high levels of engagement based on the empirical distributions of the summed scores for each domain. Finally, these ordinal activity measures were used in the subsequent LCA. The correlation matrices among continuous and ordinal activity measures are presented in Supplementary Materials Tables S1–S4.

Next, to examine factors associated with activity profiles, we utilized several individual-level socioeconomic characteristics as covariates. These include gender (1 = female, 0 = male), age (years), homeownership (1 = yes, 0 = no), marital status (1 = having a spouse, 0 = no), education (below elementary school, elementary school, middle school, high school, college or above) and income (below KRW 1 million, KRW 1–2 million, KRW 2 million or above). Self-rated health was originally measured by the Likert-type scale ranging from 1 to 5 and recoded into a three-level categorical measure (1 = poor, 2 = moderate, 3 = good).

### 3.3. Analytic Procedures

This study utilized latent class analysis (LCA) to empirically determine discrete latent classes from a series of observed activity measures and to create subgroups of older adults that share similar engagement patterns [61]. After extracting latent classes that fit the data, LCA provides conditional probabilities of being in each class for each observation and, to characterize each class, produces probabilities of engaging in each activity for each class [62,63]. In this study, we consider the LCA model with *r* observed ordinal activity items *u* with a categorical latent variable *c* with *k* classes (*c* = *k*; *k* = *1*, *2*, …, *K*). The marginal item probability for item *u_j_* = 1 is

(1) $$P\left(u_{j}=1\right)=\overset{K}{\underset{k=1}{\sum }}P\left(c=k\right)P(u_{j}=1|c=k)$$

Assuming conditional independence, the joint probability of all *r* observed items is

(2) $$P\left(u_{1},u_{2},\ldots ,u_{r}\right)=\overset{K}{\underset{k=1}{\sum }}P\left(u_{1}|c=k\right)P\left(u_{2}=1|c=k\right)\ldots P(u_{r}=1|c=k)$$

One of the issues in LCA is how to decide the number of classes; we used four different model fit statistics. First, we used the likelihood ratio chi-square test for model fit. Second, we used the Bayesian Information Criterion (BIC). Lower values of the BIC indicate better model fits and a considerable number of studies have reported that the BIC is a good indicator for class enumeration [61,63]. Third, the Lo–Mandell–Rubin (LMR) test compares the improvement in model fit between the *k*-class and (*k*−*1*)-class models. A significant test result indicates that there is a significant improvement in model fit between the *k*-class and (*k*−*1*)-class models [64]. We further reported the entropy, which is a measure of uncertainty in classification. Entropy ranges from 0 to 1, with a higher value indicating higher certainty. To examine the factors associated with activity profiles derived from LCA, we used multinomial logistic regression since the dependent variable, activity profiles, is a categorical variable with multiple levels.

**Figure 1:**
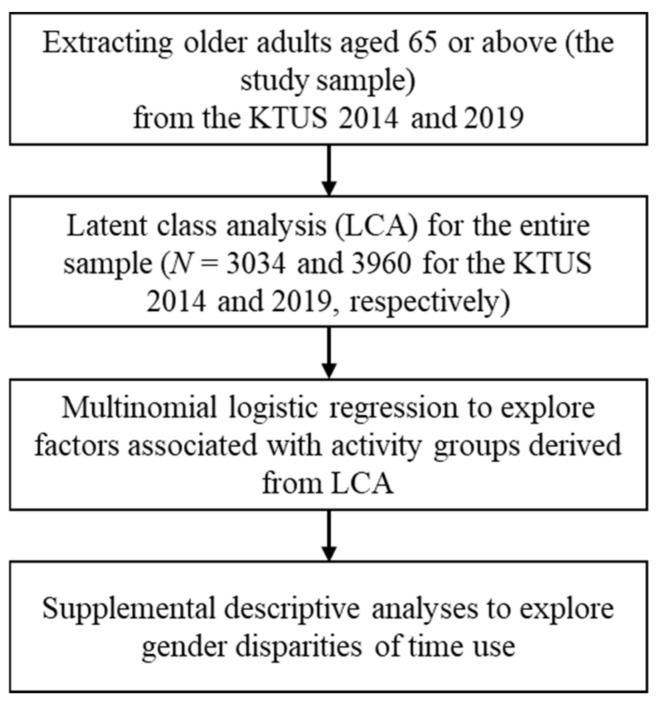
Analytic procedures.

The analytical procedures for the current study are presented in Figure 1.

## 4. Results

LCA was conducted to examine activity profiles based on a sample of Korean older adults. Model fit statistics for models with different numbers of classes are presented in Table 2. In terms of the models based on the KTUS 2014, the BIC and entropy decreased between the three- and four-class models, but the differences were small. Additionally, the LMR test indicated that the four-class model fits the data better than the three-class model. The four-class model was ultimately selected based on both model fit statistics and the substantive interpretability of the classes. For the models with the KTUS 2019, although the LMR test preferred the five-class model, the BIC decreased between the four- and five-class models. Moreover, an additional class in the five-class model did not differ much from one of the classes in the four-class model in terms of the substantive interpretability. Therefore, the four-class model was also selected for the KTUS 2019.

### 4.1. Activity Profiles among the Korean Elderly Population

**Figure 2:**
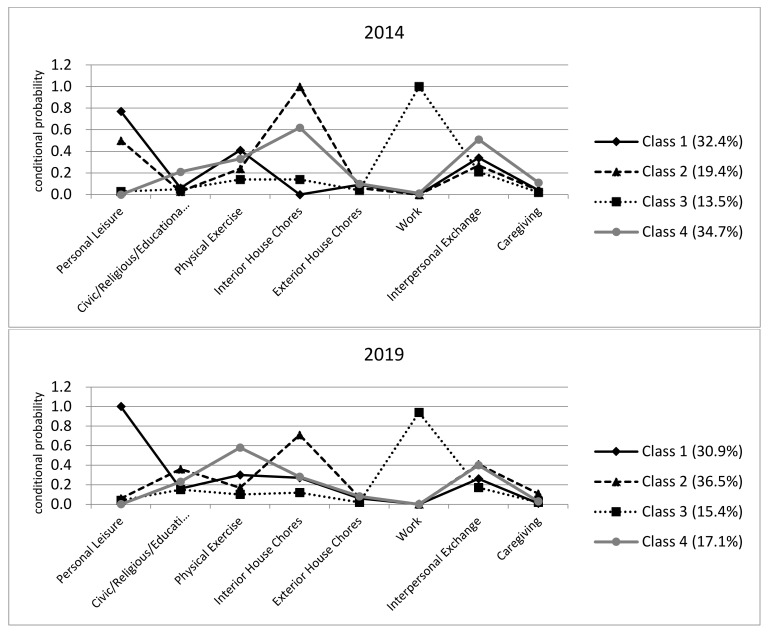
Activity profiles for Korean older adults: Findings from the LCA.

Findings from the LCA are depicted in Table 3 and Figure 2, presenting conditional probabilities of having “high” activity engagement in each activity domain.

In terms of the findings from LCA with the KTUS 2014, the study sample was distributed reasonably across the four classes. Korean older adults in Class 1, comprising 32.4% of the sample, were more likely to engage in personal leisure, physical exercise and exterior household chores. Conditional probabilities were lowest for interior household chores and work, and those for interpersonal exchanges, caregiving and civic/religious/educational activities were moderate-to-low compared with the probabilities for other groups. Older adults in Class 2 (19.4% of the sample) were characterized by the highest engagement in interior household chores and moderate engagement in personal leisure, physical exercise, exterior household chores, interpersonal exchanges and caregiving. The probabilities of engaging in work and civic/religious/educational activities were lowest for this class among the four classes. Next, Korean older adults included in Class 3 (13.5% of the sample) were most likely to engage in paid work. Engagement in other activities was considerably low. For observations in Class 4 (34.7% of the sample), the probabilities of engaging in civic/religious/educational activities, exterior household chores, interpersonal exchanges and caregiving were highest. Engagement in other activities was moderate-to-high except for activities related to personal leisure and work.

It might not be possible to clearly label each class based on the characteristics discussed above. However, for ease of discussion, we label Class 1 as “Passive–Physical Exercise, Class 2 as “Housework–Passive”, Class 3 as “Working” and Class 4 as “Nonworking–Active”.

Turning to the findings from LCA with the KTUS 2019, older adults in Class 1, comprising 30.9% of the sample, were most likely to engage in personal leisure and conditional probabilities were moderate-to-low for other activity domains. Older adults in Class 2 (36.5% of the sample) were characterized by the highest engagement in interior household chores and caregiving. One notable finding includes older adults in Class 2 also had highest probabilities of “high” engagement in civic/religious/educational activities and interpersonal exchange, compared to those in other classes. Korean older adults in Class 3, comprising 15.4% of the sample, were most likely to engage in paid work. Probabilities of engaging in other activity domains were lowest across classes as in the case of Class 3 from the KTUS 2014. Older adults in Class 4 (17.1% of the sample) were characterized by highest probabilities of engaging in physical activities and exterior household chores, and high-to-moderate probabilities of engaging in interpersonal exchange and civic/religious/educational activities.

Again, it is a difficult task to clearly label each class; however, for ease of discussion, we label Class 1 as “Passive–Personal Leisure”, Class 2 as “Housework–Active”, Class 3 as “Working” and Class 4 as “Physical Exercise–Social”. Average time spent on each activity domain across classes derived from LCA are presented in Table 4.

Next, socioeconomic characteristics of Korean older adults included in each class are presented in Table 5 and Table 6. Among others, gender disparities in class memberships are noteworthy. For example, about 85% of older adults included in Class 2 (Housework–Passive) and about 79% of older adults in Class 4 (Nonworking–Active) from the KTUS 2014 were female. Over 80% of older adults in Class 2 (Housework–Active) from the KTUS 2019 were female. Male older adults were over-represented in Class 1 (Passive–Physical Exercise) and Class 3 (Working) in 2014 and Class 3 (Working) and Class 4 (Physical Exercise–Social) in 2019.

### 4.2. Factors Associated with Activity Profiles

Findings from the multinomial logistic regression are presented in Table 7 (KTUS 2014) and Table 8 (KTUS 2019). Since one of the main purposes of these analyses is to explore whether gender is associated with activity profiles from LCA, and female older adults were over-represented in Class 2, Class 2 (Housework–Passive for 2014 and Housework–Active for 2019) was utilized as the reference group.

According to Table 7, female older adults were more likely to be in the Housework–Passive group than any other groups. Age was also a significant antecedent in two of the three models. The older elderly were more likely to be in Passive–Physical Exercise and less likely to be in Working. Older adults who were homeowners were more likely to be in Nonworking–Active than Housework–Passive. Older adults who had spouses were more likely to be in Housework–Passive than Passive–Physical Exercise or Nonworking–Active. Education and income distinguished members of Working from members of Housework–Passive, but in opposite directions; that is, older adults with lower education and higher income were more likely to be in Working.

Turning to Table 8 presenting findings from the KTUS 2019, similar patterns were found. Female older adults were more likely to be in the Housework–Active group than any other groups. Older adults who had spouses were more likely to be in Household–Active than Passive–Personal Leisure or Physical Exercise–Social. Older adults with lower education or higher income were more likely to be in the Working group. 

Table 9 presents average time spent for each activity domain for male and female older adults. During the period of 2014–2019, Korean older adults spent slightly less time for personal leisure, interior household chores and interpersonal exchange. One notable finding includes average time spent for civic/religious/educational activities increased from 24 to 40 min per day. Findings presented in Table 9 clearly show gender disparities in time use across various activity domains. For example, female older adults spent their times for interior household chores approximately 3.2 times higher than male counterparts did in 2014. In 2019, these gender differences slightly decreased although female older adults still spent their times on interior household chores approximately 2.7 times higher than male older adults. Male older adults spent more times for paid work, personal leisure and physical exercise than female counterparts did, but the opposite was true for civic/religious/educational activities and interpersonal exchange.

## 5. Discussion

This study empirically identified activity profiles for Korean older adults using LCA and further examined socioeconomic factors associated with the activity profiles derived from LCA. Gender dimensions of the activity profiles were explored in-depth.

This study revealed that Korean older adults can be classified into four distinctive groups in terms of their varying patterns of activity engagement. In 2014, Korean older adults were classified into four activity groups: Passive–Physical Exercise (32.4%), Housework–Passive (19.4%), Working (13.5%) and Nonworking Active (34.7%). In 2019, Korean older adults were also classified into four distinct activity groups although differences were found between the two waves: Passive–Personal Leisure (30.9%), Housework–Active (36.5%), Working (15.4%), Physical Exercise–Social (17.1%). These findings clearly showed that Korean older adults are not a homogeneous group; rather, there are substantial dissimilarities in terms of activity patterns.

While this study replicates and builds on prior studies, several differences in findings regarding activity patterns are noteworthy. The Working groups were consistently identified as in other studies, but the proportion of older adults in the Working group increased between 2014 and 2019. Further, there was no such group as the High Activity group in which older adults were highly engaged in most activity domains. The findings also suggest that there is some heterogeneity in activity patterns among Korean older adults between 2014 and 2019, although same activity measures and statistical procedures were used. These differences may reflect changes in activity engagement and social contexts over time in Korea as discussed in the previous section. These points are to be discussed in more detail below.

While caution should be exercised in interpreting these findings in comparison with studies from Western countries such as the United States [11,15,22], Korean older adults seemed to engage less in productive activities such as civic/religious/educational activities and more in passive activities such as personal leisure. Morrow-Howell and her colleagues found that older adults in the United States can be grouped into five distinct groups, and one of the five groups was the High Activity group, characterized by high engagement in most activity domains except paid work [11,15]. The High Activity group may be the model of “active retirement” [15], but there was no such comparable group in Korea. Another notable finding includes that probabilities of engaging in housework were more evenly distributed across activity groups among the U.S. elderly than their Korean counterparts [15]. However, there seemed to be changes between 2014 and 2019 among the Korean older adults, in that probabilities of engaging in housework were more evenly distributed across activity groups in 2019 than in 2014, and the Housework–Passive group did not exist in 2019.

The members of the Working groups in both 2014 and 2019 were low on most activities except paid work, indicating they might spend less time for managing their personal lives and other productive activities for the sake of devoting more time to work. While the role-substitution hypothesis argues that retirement or leaving paid work might increase other productive activities because of changes in the status of individuals [29,41,42,65], it is important to understand that work cessation in later life may not automatically increase activities in other domains [15]. Programs and policies should pay attention to how this group of older adults could increase engagement in productive activities after leaving paid work.

Approximately 34% and 36% of the Korean elderly in the study sample were included in the Nonworking–Active group in 2014 and in the Housework–Active group in 2019, which were the largest groups of the four activity groups derived from LCA in 2014 and 2019, respectively. Members in these two groups were high on several “productive” activities such as civic/educational/religious activities, caregiving or others. These findings at least partly support the role-enhancement hypothesis, which asserts that certain types of caregiving could enhance other productive activities, such as volunteering or informal helping [48].

In terms of socioeconomic factors associated with activity profiles, gender dimensions seemed to play a major role in explaining varying patterns of activities. Female older adults were more likely to be included in the Housework–Passive group in 2014 and the Housework–Active group in 2019 than their male counterparts, and these findings are consistent with previous studies conducted in Korea [34,35,55] as well as those in other countries [66,67]. The findings also confirmed that stable economic conditions might be associated with activity engagement among older adults, reflected in the fact that family assets such as homeownership were positively associated with being in the Nonworking–High Activity group in 2014. Consistent with the prior literature [68], family structures also play an important role. Korean older adults with spouses consistently showed higher conditional probabilities of being in the Housework–Passive group in 2014 and the Housework–Active group in 2019.

Gender disparities in activity profiles are confirmed by average times spent on each activity domain between male and female older adults. Female older adults spent their times for interior household chores approximately 3.2 times higher than male counterparts did in 2014. In 2019, these gender differences slightly decreased although female older adults still spent their times on interior household chores approximately 2.7 times higher than male older adults, indicating that the burden of unpaid housework and caregiving was still shouldered by female older adults [32,38,39]. Male older adults spent more times for paid work, personal leisure and physical exercise than female counterparts, but the opposite was true for civic/religious/educational activities and interpersonal exchange. These findings clearly suggest that programs and policies to enhance activity engagement among older adults should deliberately consider gender disparities in activity engagement.

Before further discussing the theoretical and policy implications of this study, a few limitations should be noted. First, this study used two most recent waves of cross-sectional data to empirically explore activity profiles as well as factors associated with them. More in-depth studies based on longitudinal data are needed. Further, relationships between activity profiles and well-being outcomes might need to be investigated for the Korean elderly sample in future studies. Second, largely due to data limitations, this study could not incorporate a wide range of factors potentially associated with activity profiles into the analytic model. If data on a wide range of activities based on the time diary method are included in the existing longitudinal data like the Health and Retirement Study (HRS) in the United States, complex relationships among activity profiles, antecedents and well-being outcomes can be explored in-depth in future studies. Third, this study only included older adults living in urban area as the study sample since there could be differences between older adults living in urban and rural areas in terms of activities and the contexts related to them. In the same vein, this study only analyzed time use from weekdays to capture daily activities among older adults. Further studies may need to be conducted to address differences in activity profiles and related factors between weekdays and weekends as well as those between urban and rural areas. Last, but not least, future studies may explore activity profiles of older adults in a cross-national, comparative perspectives. Cross-national contexts related to varying patterns of activity profiles may include, but not limited to, social and cultural norms, labor market situations, social policies for older adults, health and dietary factors and so on.

## 6. Conclusions 

This study contributes to the current literature by adding empirical evidence from the Korean elderly population based on broader perspectives on activities in later life. The findings from this study can also inform policy-makers as they develop policies and programs to promote the well-being of older adults through activities. While policy contexts do not alter time constraints for activity engagement among older adults, they could influence constraints from income, health and family responsibility, as well as opportunities for activity engagement [54].

This study revealed that Korean older adults seemed to engage less in “productive” activities such as civic, religious and educational activities than those from Western countries. These findings call for the initiation of well-designed policies where opportunities for activity engagement can be encouraged for this segment of the elderly. Productive or active aging has become a prominent topic of rhetoric in Korea, but few resources have been devoted to it beyond the labor market. Korean social policy for elderly adults tends to focus on “making the elderly work”, including but not limited to raising the retirement age and creating incentives to remain in the labor market longer. Many older adults also engage in paid work in publicly provided or subsidized short-term, low-income jobs. This policy focus should be complemented by a broader, comprehensive approach including continued participation in a wide range of productive activity domains, such as volunteering, education, training, leisure, recreation and other types of social participation. In addition to providing opportunities for activity engagement, policies also need to be directed at fostering social conditions and infrastructures such as transportation, public spaces and leisure facilities for the elderly as well as developing a socially cohesive community [7].

This study also revealed that stable economic conditions are closely related to increased activity engagement. It is well known that elderly poverty rates are the highest in Korea among OECD countries [69]. There is a concern that the economic downturn coupled with already high elderly poverty rates may result in a loss of opportunities for activity. These structural and economic barriers to activity engagement should be addressed for a comprehensive approach to work in practice.

This study clearly showed gender disparities in terms of activity engagement among Korean older adults. As discussed earlier, macro-level factors such as social policy, social norms for gender roles and cultural contexts contribute to this disparity. Given the gender imbalance in activity profiles, policy-makers may introduce or develop policies to relieve the burden of housework and caregiving placed on elderly women. Further, it is necessary to build a sociocultural foundation to narrow the gender gap in activity engagement.

## Figures and Tables

**Table 1:** Types and amount of activities: KTUS 2014 and 2019.

Activity Domains	Activity Items	2014		2019	
		Participants ^a^ *N* (%)	Time Spent ^b^ M (SD)	Participants ^a^ *N* (%)	Time Spent ^b^ M (SD)
Personal Leisure	Watching TV/movies/concerts/theater/sports, reading books/newspapers/magazines, listening to radio/music, searching the internet, playing games, other personal leisure activities	2958 (97.5%)	278.4 (147.6)	3824 (96.6%)	260.5 (146.9)
Civic/Religious/ Educational activity	Education at schools or other institutions, other educational activities, social participation, volunteering, helping others, religious activities	612 (20.2%)	120.2 (97.3)	2107 (53.2%)	78.0 (77.6)
Physical exercise	Walking, running, climbing, playing sports, other physical activities	1603 (52.8%)	94.2 (63.8)	1773 (55.2%)	90.4 (62.0)
Interior household chores	Preparing meals, washing dishes, other meal-related activities, laundry, cleaning, shopping, other interior household chores	2544 (83.8%)	156.2 (109.8)	3318 (83.8%)	149.5 (103.6)
Exterior household chores	Vehicle maintenance, pet care, home improvement, yard work, other exterior household chores	410 (13.5%)	37.9 (34.3)	547 (13.8%)	43.2 (36.3)
Paid Work	Employed or self-employed, unpaid family business, other work-related activities	844 (27.8%)	256.7 (193.2)	887 (22.4%)	323.7 (189.1)
Interpersonal exchange	Socializing with others, other interpersonal activities	2082 (68.3%)	75.3 (58.9)	2540 (64.1%)	71.3 (57.4)
Caregiving	Taking care of spouse, children or other relatives, other caregiving activities	364 (12.0%)	72.8 (81.0)	433 (10.9%)	77.6 (79.4)

Note: ^a^. Number and proportion (%) of participants who have non-zero minutes for each activity domain. ^b^. Average time spent and standard deviations for those who participated in activities (measured as minutes per day).

**Table 2:** Model fit statistics for LCA for male and female older adults.

	KTUS 2014 (*N* = 3034)				KTUS 2019 (*N* = 3960)			
	LRχ^2^ Test	BIC	Entropy	LMR Test	LRχ^2^ Test	BIC	Entropy	LMR Test
1-class	3710 (df = 6538)	40,351	-	-	4660 (df = 6542)	55,094	-	-
2-class	2764 (df = 6517)	39,583	1.00	897.92 ***	3350 (df = 6524)	53,940	0.98	1285.75 ***
3-class	2170 (df = 6499)	39,136	0.86	579.51 ***	2713 (df = 6507)	53,445	0.84	632.18 ***
4-class	2082 (df = 6483)	39,174	0.82	96.78 **	2560 (df = 6488)	53,452	0.78	132.59 ***
5-class	-	-	-	-	2482 (df = 6473)	53,495	0.75	96.44 **
6-class	-	-	-	-	2412 (df = 6456)	53,566	0.75	69.98

** *p* < 0.01, *** *p* < 0.001; BIC = Bayesian Information Criterion; LMR = Lo–Mendell–Rubin; Model fit statistics for the five-class model with the KTUS 2014 were not presented due to non-convergence.

**Table 3:** Conditional probabilities of being in “high” activity engagement.

	2014				2019			
	Class 1	Class 2	Class 3	Class 4	Class 1	Class 2	Class 3	Class 4
Personal Leisure	0.77	0.50	0.03	0.00	1.00	0.06	0.04	0.00
Civic/Religious/Educational Activity	0.06	0.03	0.05	0.21	0.16	0.36	0.15	0.23
Physical Exercise	0.41	0.24	0.14	0.33	0.30	0.17	0.10	0.58
Interior Household Chores	0.00	1.00	0.14	0.62	0.27	0.71	0.12	0.28
Exterior Household Chores	0.09	0.06	0.05	0.10	0.06	0.07	0.02	0.08
Paid Work	0.00	0.00	1.00	0.01	0.00	0.00	0.94	0.00
Interpersonal Exchange	0.34	0.27	0.21	0.51	0.26	0.41	0.17	0.40
Caregiving	0.04	0.04	0.02	0.11	0.01	0.11	0.02	0.03

**Table 4:** Average time spent on each activity domain (measured as minutes per day).

2014	2014				2019			
	Passive–Physical Exercise	Housework–Passive	Working	Nonworking–Active	Passive–Personal Leisure	Housework–Active	Working	Physical Exercise–Social
Personal Leisure	403	348	124	171	425	164	114	220
Civic/Religious/Educational Activity	9	8	10	51	27	61	23	38
Physical Exercise	67	39	23	49	52	30	21	119
Interior Household Chores	47	230	58	192	93	202	52	80
Exterior Household Chores	5	5	3	6	5	7	2	7
Paid Work	12	6	417	23	5	16	421	17
Interpersonal Exchange	48	28	29	74	37	59	24	54
Caregiving	5	5	2	16	3	18	2	2

**Table 5:** Characteristics of Korean older adults by classes (KTUS 2014).

		Total	Passive– Physical Exercise	Houework–Passive	Working	Nonworking–Active
		*N* (%)	*N* (%)	*N* (%)	*N* (%)	*N* (%)
Gender	Male	1221 (40.2)	669 (65.6)	73 (14.8)	249 (59.3)	230 (21.0)
	Female	1813 (59.8)	352 (34.5)	422 (85.2)	171 (40.7)	868 (79.0)
Home- owner	Yes	2270 (74.8)	749 (73.4)	357 (72.1)	307 (73.1)	857 (78.0)
	No	764 (25.2)	272 (26.6)	138 (27.9)	113 (26.9)	241 (22.0)
Spouse	Yes	1758 (57.9)	634 (62.1)	262 (52.9)	296 (70.5)	566 (51.6)
	No	1276 (42.1)	387 (37.9)	233 (47.1)	124 (29.5)	532 (48.4)
Education	Below elementary school	768 (25.3)	231 (22.6)	155 (31.3)	77 (18.3)	305 (27.8)
	Elementary school	968 (31.9)	310 (30.4)	180 (36.4)	105 (25.0)	373 (34.0)
	Middle school	497 (16.4)	161 (15.8)	72 (14.6)	99 (23.6)	165 (15.0)
	High school	547 (18.0)	200 (19.6)	64 (12.9)	93 (22.1)	190 (17.3)
	College or above	254 (8.4)	119 (11.7)	24 (4.8)	46 (11.0)	65 (5.9)
Income	Below KRW 1 million	2334 (76.9)	767 (75.1)	446 (90.1)	187 (44.5)	934 (85.1)
	KRW 1–2 million	454 (15.0)	156 (15.3)	27 (5.5)	157 (37.4)	114 (10.4)
	KRW 2 million or above	246 (8.1)	98 (9.6)	22 (4.4)	76 (18.1)	50 (4.6)
Self-rated health	Poor	857 (28.2)	313 (30.7)	157 (31.7)	75 (17.9)	312 (28.4)
	Moderate	1371 (45.2)	447 (43.8)	232 (46.9)	207 (49.3)	485 (44.2)
	Good	806 (26.6)	261 (25.6)	106 (21.4)	138 (32.9)	301 (27.4)
		**M (SD)**	**M (SD)**	**M (SD)**	**M (SD)**	**M (SD)**
Age		73.81 (6.38)	75.35 (6.59)	73.96 (6.12)	69.84 (4.40)	73.83 (6.28)

**Table 6:** Characteristics of Korean older adults by classes (KTUS 2019).

		Total	Passive– Personal Leisure	Housework–Active	Working	Physical Exercise–Social
		*N* (%)	*N* (%)	*N* (%)	*N* (%)	*N* (%)
Gender	Male	1699 (42.9)	679 (51.9)	281 (19.5)	367 (62.3)	372 (60.2)
	Female	2261 (57.1)	630 (48.1)	1163 (80.5)	222 (37.7)	246 (39.8)
Home- owner	Yes	3015 (76.1)	983 (75.1)	1097 (76.0)	463 (78.6)	472 (76.4)
	No	945 (23.9)	326 (24.9)	347 (24.0)	126 (21.4)	146 (23.6)
Spouse	Yes	2433 (61.4)	782 (59.7)	808 (56.0)	458 (77.8)	385 (62.3)
	No	1527 (38.6)	527 (40.3)	636 (44.0)	131 (22.2)	233 (37.7)
Education	Below elementary school	728 (18.4)	284 (21.7)	309 (21.4)	44 (7.5)	91 (14.7)
	Elementary school	1156 (29.2)	386 (29.5)	471 (32.6)	143 (24.3)	156 (25.2)
	Middle school	765 (19.3)	216 (16.5)	275 (19.0)	135 (22.9)	139 (22.5)
	High school	910 (23.0)	282 (21.5)	290 (20.1)	190 (32.3)	148 (24.0)
	College or above	401 (10.1)	141 (10.8)	99 (6.9)	77 (13.1)	84 (13.6)
Income	Below KRW 1 million	3072 (77.6)	1116 (85.3)	1279 (88.6)	179 (30.4)	498 (80.6)
	KRW 1–2 million	499 (12.6)	98 (7.5)	102 (7.1)	237 (40.2)	62 (10.0)
	KRW 2 million or above	389 (9.8)	95 (7.3)	63 (4.4)	173 (29.4)	58 (9.4)
Self-rated health	Poor	770 (19.4)	288 (22.0)	316 (21.9)	64 (10.9)	102 (16.5)
	Moderate	2241 (56.6)	746 (57.0)	810 (56.1)	356 (60.4)	329 (53.2)
	Good	949 (24.0)	275 (21.0)	318 (22.0)	169 (28.7)	187 (30.3)
		**M (SD)**	**M (SD)**	**M (SD)**	**M (SD)**	**M (SD)**
Age		74.01 (6.71)	75.80 (6.93)	73.92 (6.39)	69.54 (4.56)	74.71 (6.70)

**Table 7:** Socioeconomic factors associated with activity groups (KTUS 2014).

2014		Passive– Physical Exercise vs. Houework–Passive	Working vs. Houework–Passive	Nonworking–Active vs. Houework–Passive
		EST (SE)	EST (SE)	EST (SE)
Gender	(Male)			
	Female	−2.71 (0.17) ***	−1.72 (0.20) ***	−0.38 (0.17) *
Age		0.05 (0.01) ***	−0.12 (0.01) ***	0.0002 (0.01)
Homeowner	(No)			
	Yes	−0.04 (0.14)	−0.23 (0.17)	0.34 (0.13) **
Spouse	(No)			
	Yes	−0.56 (0.15) ***	−0.24 (0.18)	−0.32 (0.13) *
Education	(Below elementary school)			
	Elementary school	−0.02 (0.16)	−0.67 (0.21) ***	0.08 (0.14)
	Middle school	−0.04 (0.21)	−0.50 (0.25) *	0.18 (0.19)
	High school	−0.06 (0.22)	−0.98 (0.27) ***	0.34 (0.20) +
	College or above	0.11 (0.31)	−1.07 (0.36) **	0.26 (0.30)
Income	(Below KRW 1 million)			
	KRW 1–2 million	0.55 (0.23) *	2.05 (0.24) ***	0.49 (0.23) *
	KRW 2 million or above	−0.08 (0.29)	1.29 (0.31) ***	−0.26 (0.29)
Self-rated health	(Poor)			
	Moderate	−0.26 (0.14) +	0.22 (0.18)	−0.01 (0.13)
	Good	−0.12 (0.16)	0.31 (0.21)	0.26 (0.15) +
Constant		−0.68 (0.86)	10.08 (1.17) ***	0.81 (0.80)

*N* = 3034; LR χ^2^ (df = 36) = 1169.84 ***; Pseudo R^2^ = 0.15; + *p* < 0.10, * *p* < 0.05, ** *p* < 0.01, *** *p* < 0.001.

**Table 8:** Socioeconomic factors associated with activity groups (KTUS 2019).

2019		Passive– Personal Leisure vs. Housework–Active	Working vs. Housework–Active	Physical Exercise–Social vs. Housework–Active
		EST (SE)	EST (SE)	EST (SE)
Gender	(Male)			
	Female	−1.64 (0.10) ***	−1.20 (0.14) ***	−1.91 (0.12) ***
Age		0.04 (0.01) ***	−0.10 (0.01) ***	0.03 (0.01) **
Homeowner	(No)			
	Yes	−0.07 (0.10)	−0.11 (0.14)	0.001 (0.12)
Spouse	(No)			
	Yes	−0.21 (0.10) *	0.10 (0.15)	−0.42 (0.12) **
Education	(Below elementary school)			
	Elementary school	−0.04 (0.12)	−0.04 (0.21)	0.11 (0.16)
	Middle school	−0.22 (0.14)	−0.20 (0.23)	0.30 (0.18) +
	High school	−0.09 (0.14)	−0.38 (0.23)	0.18 (0.18)
	College or above	0.07 (0.19)	−0.81 (0.27) **	0.39 (0.23) +
Income	(Below KRW 1 million)			
	KRW 1–2 million	−0.17 (0.16)	2.33 (0.15) ***	−0.02 (0.18)
	KRW 2 million or above	0.002 (0.19)	2.32 (0.20) ***	0.001 (0.22)
Self-rated health	(Poor)			
	Moderate	−0.01 (0.10)	0.27 (0.17)	0.14 (0.14)
	Good	−0.13 (0.13)	0.13 (0.20)	0.39 (0.01) *
Constant		−1.77 (0.58) **	6.32 (0.96) ***	−1.66 (0.73)

*N* = 3960; LR χ^2^ (df = 36) = 1489.83 ***; Pseudo R^2^ = 0.14; + *p* < 0.10, * *p* < 0.05, ** *p* < 0.01, *** *p* < 0.001.

**Table 9:** Average time spent for each activity domain by gender (measured as minutes per day).

	2014			2019		
	Total	Male	Female	Total	Male	Female
Personal Leisure	271	307	247	252	277	232
Civic/Religious/Educational Activity	24	15	31	40	30	49
Physical Exercise	50	69	37	50	69	35
Interior Household Chores	131	57	181	125	64	171
Exterior Household Chores	5	6	4	6	7	6
Paid Work	71	103	50	73	106	47
Interpersonal Exchange	52	42	58	46	38	51
Caregiving	9	8	9	8	7	9

## Data Availability

The KTUS datasets are available from the MicroData Integrated Service (MDIS), Statistics Korea (https://mdis.kostat.go.kr/, accessed on 25 June 2021).

## Supplementary Materials

The following are available online at https://www.mdpi.com/article/10.3390/ijerph18168786/s1, Table S1: Pearson correlation matrix among activity domains (continuous activity indicators): KTUS 2014; Table S2: Pearson correlation matrix among activity domains (continuous activity indicators): KTUS 2019; Table S3: Polychoric correlation matrix among activity domains (ordinal activity indicators): KTUS 2014; Table S4. Polychoric correlation matrix among activity domains (ordinal activity indicators): KTUS 2019.

## References

[1] Statistics Korea (2019). Population Projections for Korea: 2017~2067.

[2] Steinmayr D., Weichselbaumer D., Winter-Ebmer R. (2019). Gender Differences in Active Ageing: Findings from a New Individual-Level Index for European Countries.

[3] World Health Organization (2002). Active Ageing: A Policy Framework.

[4] Lum T.Y. (2013). Advancing research on productive aging activities in greater Chinese societies. Ageing Int..

[5] Schulte P.A., Grosch J., Scholl J.C., Tamers S.L. (2018). Framework for considering productive aging and work. J. Occup. Environ. Med..

[6] Sherraden M., Hinterlong J., Morrow-Howell N. (2001). Productive Aging: Concepts and Challenges.

[7] Foster L., Walker A. (2013). Gender and active ageing in Europe. Eur. J. Ageing.

[8] Nillson H., Bulow P., Kazemi A. (2015). Mindful sustainable aging: Advancing a comprehensive approach to the challenges and opportunities of old age. Eur. J. Psychol..

[9] Gonzales E., Matz-Costa C., Morrow-Howell N. (2015). Increasing opportunities for the productive engagement of older adults: A response to population aging. Gerontologist.

[10] Buchman A.S., Wilson R.S., Bennett D.A. (2008). Total daily activity is associated with cognition in older persons. Am. J. Geriatr. Psychiatry.

[11] Chen Y., Putnam M., Lee Y., Morrow-Howell N. (2019). Activity patterns and health outcomes in later life: The role of nature of engagement. Gerontology.

[12] Gil-Lacruz M., Saz-Gil M.I., Gil-Lacruz A.I. (2019). Benefits of older volunteering on well-being: International comparison. Front. Psychol..

[13] Glass T.A., Mendes de Leon C.M., Bassuk S.S., Berkman L.F. (2006). Social engagement and depressive symptoms in late life. J. Aging Health.

[14] Janke M.C., Payne L.L., Van Puymbroeck M. (2008). The role of informal and formal leisure activities in the disablement process. Int. J. Aging Hum. Dev..

[15] Morrow-Howell N., Putnam M., Lee Y., Greenfield J.C., Inoue M., Chen H. (2014). An investigation of activity profiles of older adults. J. Gerontol. Ser. B Psychol. Sci. Soc. Sci..

[16] Paggi M.E., Jopp D., Hertzog C. (2016). The importance of leisure activities in the relationship between physical health and well-being in a life span sample. Gerontology.

[17] Silverwood R.J., Nitsch D., Pierce M., Kuh D., Mishra G.D. (2011). Characterizing longitudinal patterns of physical activity in mid-adulthood using latent class analysis: Results from a prospective cohort study. Am. J. Epidemiol..

[18] Janke M.C., Davey A., Kleiber D. (2006). Modeling change in older adults’ leisure activities. Leis. Sci..

[19] Litzwin H., Shiovitz-Ezra S. (2006). The association between activity and wellbeing in later life: What really matters?. Ageing Soc..

[20] Jopp D.S., Herzog C. (2010). Assessing adult leisure activities: An extension of a self-report activity questionnaire. Psychol. Assess..

[21] Baker L.A., Silverstein M. (2008). Depressive symptoms among grandparents raising grandchildren: The impact of participation in multiple roles. J. Intergener. Relatsh..

[22] Burr J.A., Mutchler J.E., Caro F.G. (2007). Productive activity clusters among middle-aged and older adults: Intersecting forms and time commitments. J. Gerontol. Ser. B Psychol. Sci. Soc. Sci..

[23] Dosman D., Fast J., Chapman S.A., Keating N. (2006). Retirement and productive activity in later life. J. Fam. Econ. Issue.

[24] Fast J.E., Dosman D., Moran L. (2006). Productive activity in later life. Res. Aging.

[25] Hinterlong J.E. (2008). Productive engagement among older Americans: Prevalence, patterns, and implications for public policy. J. Aging Soc. Policy.

[26] Sugihara Y., Sugisawa H., Shibata H., Harada K. (2008). Productive roles, gender, and depressive symptoms: Evidence from a national longitudinal study of late-middle-aged Japanese. J. Gerontol. Ser. B Psychol. Sci. Soc. Sci..

[27] Arai A., Ishida K., Tommori M., Katsumata Y., Grove J., Tamashiro H. (2007). Association between lifestyle activity and depressed mood among home-dwelling older people: A community-based study in Japan. Aging Ment. Health.

[28] Bielak A.A.M., Hughes T.F., Small B.J., Dixon R.A. (2007). It’s never too late to engage in lifestyle activities: Significant concurrent but not change relationships between lifestyle activities and cognitive speed. J. Gerontol. Ser. B Psychol. Sci. Soc. Sci..

[29] Liu H., Lou W.Q. (2016). Patterns of productive activity engagement among older adults in urban China. Eur. J. Ageing.

[30] Morrow-Howell N., Hong S., McCrary S., Blinne W. (2012). Changes in activity among older volunteers. Res. Aging.

[31] Paillard-Borg S., Wang H., Winblad B., Fratiglioni L. (2009). Pattern of participation in leisure activities among older people in relation to their health conditions and contextual factors: A survey in a Swedish urban area. Ageing Soc..

[32] Chung S., Lee E. (2017). Patterns of time use across the life span in Korea: A latent class analysis and age and gender differences. Soc. Indic. Res..

[33] Kim J. (2019). Productive aging of Korean older people based on time use. Soc. Sci. Med..

[34] Kim J. (2006). A study on the time use of the elderly in Korea: Analyzing their use of time upon work, family and leisure. J. Welf. Aged.

[35] Park S. (2007). A study on the gender gap in the Korean elderly women’s time use. Women’s Stud..

[36] Torres S. (1999). A culturally-relevant theoretical framework for the study of successful ageing. Ageing Soc..

[37] Burda M., Hamermesh D., Weil P. (2013). Total work and gender: Facts and possible explanations. J. Popul. Econ..

[38] Huh S., Kim H. (2019). Time use and division of housework in dual-earner households in Korea. Korean J. Fam. Welf..

[39] Kim Y. (2014). A comparative study on the relation between welfare state policy and unpaid work time by gender. Korean Soc. Policy Rev..

[40] Lum T.Y., Lightfoot E. (2005). The effects of volunteering on the physical and mental health of older people. Res. Aging.

[41] Hank K., Stuck S. (2008). Volunteer work, informal help, and care among the 50+ in Europe: Further evidence for ‘linked’ productive activities at older ages. Soc. Sci. Res..

[42] Mutchler J.E., Burr J.A., Caro F.G. (2003). From paid worker to volunteer: Leaving the paid workforce and volunteering in later life. Soc. Forces.

[43] Robinson J.P., Godbey G. (2000). Time for Life: The Surprising Ways Americans Use Their Time.

[44] Carr D.C., Kail B.L. (2013). The influence of unpaid work on the transition out of full-time paid work. Gerontologist.

[45] Jacobs J.C., Van Houtven C.V., Laporte A., Coyte P.C. (2017). The impact of informal caregiving intensity on women’s retirement in the United States. J. Popul. Ageing.

[46] Rosario P.A., Morrow-Howell N., Hinterlong J.E. (2004). Role enhancement or role strain assessing the impact of multiple productive roles on older caregiver well-being. Res. Aging.

[47] Choi N., Burr J.A., Mutchler J.E., Caro F.G. (2007). Formal and informal volunteer activity and spousal caregiving among older adults. Res. Aging.

[48] Stanfor M., Jacobs J.C., Neilson J. (2019). Caregiving time costs and trade-offs: Gender differences in Sweden, the UK, and Canada. Soc. Sci. Med. SSM Popul. Health.

[49] Van der Horst M., Vickerstaff S., Lain D., Clark C., Geiger B.B. (2017). Pathways of paid work, care provision, and volunteering in later careers: Activity substitution or extension?. Work Aging Retire..

[50] De Preter H., Van Looy D., Mortelmans D., Denaeghel K. (2013). Retirement timing in Europe: The influence of individual work and life factors. Soc. Sci. J..

[51] Carmel S. (2019). Health and well-being in late life: Gender differences worldwide. Front. Med..

[52] Lee Y., Tang F. (2015). More caregiving, less working: Caregiving roles and gender difference. J. Appl. Gerontol..

[53] Avital D. (2017). Gender differences in leisure patterns at age 50 and above: Micro and macro aspects. Ageing Soc..

[54] Gauthier A.H., Smeeding T.M. (2003). Time use at older ages: Cross-national differences. Res. Aging.

[55] An M.Y. (2017). Gender division of labour at home among older couples in South Korea. Women’s Stud..

[56] Juster F.T., Stafford F.P. (1991). The allocation of time: Empirical findings, behavioral models, and problems of measurement. J. Econ. Lit..

[57] Kan M.Y., Pudney S. (2008). Measurement error in stylized and diary data on time use. Sociol. Methodol..

[58] Song Y. (2011). Changes in parental time spent with children. Korean J. Popul. Stud..

[59] Statistics Korea (2016). The Korean Time Use Survey (KTUS) 2014: User’s Guide.

[60] Statistics Korea (2020). The Korean Time Use Survey (KTUS) 2019.

[61] Hagenaars J., McCutcheon A. (2002). Applied Latent Class Analysis.

[62] Muthen B., Muthen L. (2000). Integrating person-centered and variable-centered analysis: Growth mixture modeling with latent trajectory classes. Alcohol. Clin. Exp. Res..

[63] Nylund K.L., Asparouhov T., Muthen B.O. (2007). Deciding on the number of classes in latent class analysis and growth mixture modeling: A Monte Carlo simulation study. Struct. Equ. Modeling A Multidiscip. J..

[64] Lo Y., Mendell N.R., Rubin D.B. (2001). Testing the number of components in a normal mixture. Biometrika.

[65] Wahrendorf M., Siegrist J. (2010). Are changes in productive activities of older people associated with changes in their well-being? Results of a longitudinal European study. Eur. J. Ageing.

[66] Campana J.C., Gimenez-Nadal J.I., Molina J.A. (2015). Gender Differences in the Distribution of Total Work-Time of Latin-American Families: The Importance of Social Norms.

[67] Coltrane S. (2000). Research on household labor: Modeling and measuring the social embeddedness of routine family work. J. Marriage Fam..

[68] Choi L.H. (2003). Factors affecting volunteerism among older adults. J. Appl. Gerontol..

[69] OECD (2019). Pensions at a Glance.

