# Supplementary material for: Activity Profiles among Older Adults: Latent Class Analysis Using the Korean Time Use Survey
Source: Int J Environ Res Public Health. 2021 Aug 20;18(16):8786. doi: 10.3390/ijerph18168786 (PMC8392169; doi:10.3390/ijerph18168786)
Supplement: Supplementary file 1 [file ijerph-18-08786-s001.zip › ijerph-1292859-supplementary.pdf]

Supplementary Material

Table S1. Pearson correlation matrix among activity domains (continuous activity indicators): KTUS 2014

	F1	F2	F3	F4	F5	F6	F7	F8
F1	1.00							
F2	-0.19***	1.00						
F3	0.06**	-0.06**	1.00					
F4	-0.25***	0.03	-0.15***	1.00				
F5	-0.01	-0.04*	0.003	0.02	1.00			
F6	-0.42***	-0.10***	-0.18***	-0.26***	-0.06**	1.00		
F7	-0.12***	-0.01	-0.09***	-0.02	-0.04*	-0.14***	1.00	
F8	-0.09***	-0.01	-0.02	0.04*	-0.01	-0.07***	-0.04*	1.00

F1: personal leisure, F2: civic/religious/educational activity, F3: physical exercise, F4: interior household chores, F5: exterior household chores, F6: paid work, F7: interpersonal exchange, F8: caregiving; * p<.05 ** p<.01 *** p<.001

Table S2. Pearson correlation matrix among activity domains (continuous activity indicators): KTUS 2019

	F1	F2	F3	F4	F5	F6	F7	F8
F1	1.00							
F2	-0.19***	1.00						
F3	0.05***	-0.06***	1.00					
F4	-0.21***	0.04*	-0.14***	1.00				
F5	-0.003	0.01*	-0.003	0.03	1.00			
F6	-0.41***	-0.10***	-0.19***	-0.28***	-0.08***	1.00		
F7	-0.10***	-0.01	-0.08***	0.01	-0.02	-0.17***	1.00	
F8	-0.10***	-0.02	-0.06***	0.10***	-0.02	-0.07***	-0.02	1.00

F1: personal leisure, F2: civic/religious/educational activity, F3: physical exercise, F4: interior household chores, F5: exterior household chores, F6: paid work, F7: interpersonal exchange, F8: caregiving; * p<.05 ** p<.01 *** p<.001

Table S3. Polychoric correlation matrix among activity domains (ordinal activity indicators): KTUS 2014

	F1	F2	F3	F4	F5	F6	F7	F8
F1	1.00							
F2	-0.27***	1.00						
F3	0.12***	-0.04	1.00					
F4	-0.21***	0.15***	-0.13***	1.00				
F5	0.004	-0.05	0.10**	0.04	1.00			
F6	-0.52***	-0.20***	-0.24***	-0.30***	-0.08*	1.00		
F7	-0.13***	0.08**	-0.05*	0.04	-0.04	-0.12***	1.00	
F8	-0.16***	0.03	0.02	0.19***	-0.04	-0.18***	0.02	1.00

F1: personal leisure, F2: civic/religious/educational activity, F3: physical exercise, F4: interior household chores, F5: exterior household chores, F6: paid work, F7: interpersonal exchange, F8: caregiving; * p<.05 ** p<.01 *** p<.001

Table S4. Polychoric correlation matrix among activity domains (ordinal activity indicators): KTUS 2019

	F1	F2	F3	F4	F5	F6	F7	F8
F1	1.00							
F2	-0.17***	1.00						
F3	0.12***	-0.04*	1.00					
F4	-0.17***	0.12***	-0.10***	1.00				
F5	0.01	0.07**	0.02	0.16***	1.00			
F6	-0.59***	-0.10***	-0.26***	-0.38***	-0.21***	1.00		
F7	-0.08***	0.05*	-0.08***	0.09***	0.01	-0.23***	1.00	
F8	-0.16***	-0.01	-0.08***	0.23***	-0.03	-0.21***	0.02	1.00

F1: personal leisure, F2: civic/religious/educational activity, F3: physical exercise, F4: interior household chores, F5: exterior household chores, F6: paid work, F7: interpersonal exchange, F8: caregiving; * p<.05 ** p<.01 *** p<.001
